# BioPlexR and BioPlexPy: integrated data products for the analysis of human protein interactions

**DOI:** 10.1093/bioinformatics/btad091

**Published:** 2023-02-16

**Authors:** Ludwig Geistlinger, Roger Vargas, Tyrone Lee, Joshua Pan, Edward L Huttlin, Robert Gentleman

**Affiliations:** Center for Computational Biomedicine, Harvard Medical School, Boston, MA 02115, USA; Center for Computational Biomedicine, Harvard Medical School, Boston, MA 02115, USA; Center for Computational Biomedicine, Harvard Medical School, Boston, MA 02115, USA; Department of Cell Biology, Harvard Medical School, Boston, MA 02115, USA; Department of Cell Biology, Harvard Medical School, Boston, MA 02115, USA; Center for Computational Biomedicine, Harvard Medical School, Boston, MA 02115, USA

## Abstract

**Summary:**

The BioPlex project has created two proteome scale, cell-line-specific protein–protein interaction (PPI) networks: the first in 293T cells, including 120k interactions among 15k proteins; and the second in HCT116 cells, including 70k interactions between 10k proteins. Here, we describe programmatic access to the BioPlex PPI networks and integration with related resources from within R and Python. Besides PPI networks for 293T and HCT116 cells, this includes access to CORUM protein complex data, PFAM protein domain data, PDB protein structures, and transcriptome and proteome data for the two cell lines. The implemented functionality serves as a basis for integrative downstream analysis of BioPlex PPI data with domain-specific R and Python packages, including efficient execution of maximum scoring sub-network analysis, protein domain–domain association analysis, mapping of PPIs onto 3D protein structures and analysis of BioPlex PPIs at the interface of transcriptomic and proteomic data.

**Availability and implementation:**

The BioPlex R package is available from Bioconductor (bioconductor.org/packages/BioPlex), and the BioPlex Python package is available from PyPI (pypi.org/project/bioplexpy). Applications and downstream analyses are available from GitHub (github.com/ccb-hms/BioPlexAnalysis).

## 1 Introduction

Protein–protein interactions (PPI) are physical contacts between proteins that are essential for the function, organization and regulation of molecular processes. PPIs can be experimentally detected based on yeast two-hybrid screening (Y2H) or affinity-purification mass spectrometry (AP-MS), and computationally inferred using a variety of strategies. Although comprehensive databases of validated and predicted PPIs exist, available PPI networks remain fragmentary and provide limited information on context dependency of the contained interactions. Using a large-scale AP-MS platform for two human cell lines, the BioPlex project has recently started to explore context dependency of human PPIs at proteome scale, which revealed shared and cell-specific modules between cell lines (Huttlin *et al.*, 2021). To enable straightforward and reproducible access to the BioPlex PPI networks and facilitate integration with related data resources, we provide software packages implemented in R and Python that provide analysis capabilities for a series of downstream applications and that connect the networks with domain-specific functionality of the two major data science ecosystems.

## 2 Features

**Fig. 1.**
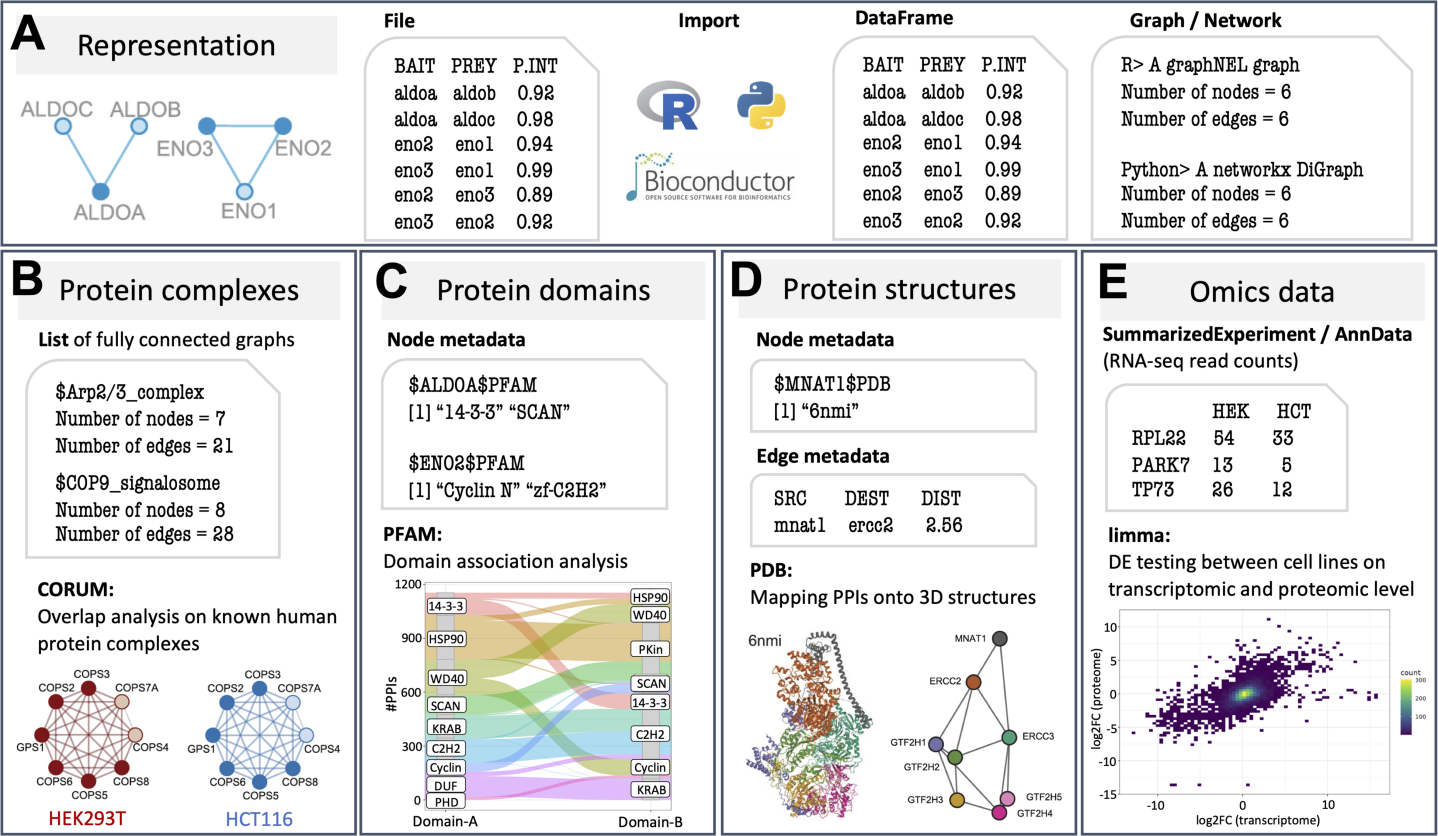
Overview. (**A**) The BioPlexR and BioPlexPy packages import PPIs from a flat file format into R and Python, and store them in dedicated graph data structures. The packages provide functionality for integration with data resources and analysis capabilities for: (**B**) overlap analysis with human protein complexes from CORUM (Giurgiu *et al*., 2019), (**C**) association analysis between protein domains from PFAM (Sonnhammer *et al*., 1998), (**D**) proximity analysis of interacting proteins in multi-chain protein structures from PDB (Berman *et al*., 2000) and (**E**) integration with transcriptomic and proteomic data for both cell lines

**Import and representation of PPI data.** The packages read PPI data from a simple file format that provide the bait ID and the prey ID for each interaction, along with a probability that the interaction resulted from a bona fide interacting partner (Fig. 1A). Once imported, the PPI data are stored for efficient representation and manipulation in dedicated graph data structures as implemented in Bioconductor’s graph package (Carey *et al.*, 2005) and Python’s NetworkX package (Hagberg *et al.*, 2008).

**Overlap analysis with human protein complexes.** For the assessment of coverage on known protein complexes, we provide functions to import complex data from CORUM (Giurgiu *et al.*, 2019). Once protein complex data have been obtained, the user can assess whether and to which extent a given PPI network overlaps with human protein complexes (Fig. 1B). As a certain amount of overlap can be expected just by chance, an assessment of statistical significance is needed to decide whether the observed overlap is greater (enrichment) or less (depletion) than expected by chance. We therefore implemented functions for testing overlaps of PPIs with a complex based on random sampling or network randomization. The random sampling procedure compares the observed number of interactions within a complex to random sub-networks of the PPI network, matching the number of subunits, the bait: prey ratio, and the node degree distribution of the complex. The network randomization procedure randomizes the network a defined number of times and calculates for each complex how often the number of edges in the complex in a randomized network exceeds the number of edges in the complex observed for the true PPI network. Both approaches can also be applied to other gene sets, and we provide visualization functions for the illustration of differential enrichment of a complex between network versions and cell lines.

**Domain–domain association analysis.** For the detection of protein domains that mediate interactions by binding complementary domains within other proteins, we provide functions to obtain protein domain information from PFAM (Sonnhammer *et al.*, 1998) and annotation to the metadata for each node of the graph (Fig. 1C). Given the obtained PFAM domains, the packages implement functionality for the identification of statistically associated domain pairs connected by a disproportionately high number of PPIs. We therefore assess domain pairs connected by two or more PPIs for significance using Fisher’s exact test based on (i) the number of PPIs connecting both domains; (ii) the numbers of PPIs involving either domain individually; and (iii) the number of PPIs not involving either domain. Although the domain association analysis identifies domain pairs whose parent proteins interact preferentially, this does not necessarily mean that these domains themselves are responsible for the interaction. Many may simply be passengers that associate as a consequence of PPIs mediated by contacts elsewhere in the protein sequence.

**Mapping PPIs onto 3D protein structures.** For the identification of PPIs that connect complex subunits or protein domains that are close enough to physically interact, we provide functionality to obtain protein structures from PDB (Berman *et al.*, 2000). As previously described (Huttlin *et al.*, 2021), we therefore map a set of proteins, for example, representing subunits of a complex, to PDB structures, and annotate the mapping to the node metadata (Fig. 1D). We then infer physical interactions within a structure by calculating the distance between the atoms of different chains within that structure. If the minimum distance between atoms of two chains is below a given threshold (default: 6 Å), these proteins are defined to interact directly; all other pairs of proteins that occurred in the same structure are assumed to interact indirectly. We provide functions to visually inspect such structurally inferred interaction graphs side-by-side with the corresponding PDB structure.

**Integration with omics data.** Two sources of variability in the detection of PPIs via AP-MS are bait and prey expression, where larger fractions of detected interactions can often be explained with higher expression of either bait or prey proteins. For the systematic assessment of AP-MS variability within the BioPlex networks, we provide functions to obtain RNA-seq data for 293T cells (Sun *et al.*, 2019) and HCT116 cells (Ghandi *et al.*, 2019) and relative protein expression data comparing 293T and HCT116 cells based on tandem mass tag analysis (Huttlin *et al.*, 2021). Datasets are provided in designated data structures as implemented in the SummarizedExperiment package in R and the anndata package in Python (Fig. 1E). Maximum subscoring network analysis can be applied for the unsupervised identification of functional modules based on differential expression between both cell lines on transcriptomic and proteomic level. In a second supervised reduction step, gene set enrichment analysis can be used to identify biological themes within a module. Resulting enriched gene sets and pathways can be visualized with an R/Shiny graph viewer, allowing the flexible overlay of node and edge metadata attributes for interactive exploration at https://ccb-rstudio-connect.hms.harvard.edu/graphviewer.

## Funding

The work was funded by NIH and grant number (U24 HG006673).

*Conflict of Interest*: none declared.
